# Putative Novel Effector Genes Revealed by the Genomic Analysis of the Phytopathogenic Fungus *Fusarium oxysporum* f. sp. *physali* (*Foph*) That Infects Cape Gooseberry Plants

**DOI:** 10.3389/fmicb.2020.593915

**Published:** 2021-01-18

**Authors:** Jaime Simbaqueba, Edwin A. Rodríguez, Diana Burbano-David, Carolina González, Alejandro Caro-Quintero

**Affiliations:** ^1^Corporación Colombiana de Investigación Agropecuaria – AGROSAVIA, Centro de Investigación Tibaitatá, Mosquera, Colombia; ^2^Department of Biology, Universidad Nacional de Colombia, Bogotá, Colombia

**Keywords:** *Fusarium oxysporum* f. sp. *physali*, cape gooseberry, effector genes, pathogenicity, vascular wilt disease

## Abstract

The vascular wilt disease caused by the fungus *Fusarium oxysporum* f. sp. *physali* (*Foph*) is one of the most limiting factors for the production and export of cape gooseberry (*Physalis peruviana*) in Colombia. A transcriptomic analysis of a highly virulent strain of *F. oxysporum* in cape gooseberry plants, revealed the presence of secreted in the xylem (SIX) effector genes, known to be involved in the pathogenicity of other *formae speciales* (ff. spp.) of *F. oxysporum*. This pathogenic strain was classified as a new f. sp. named *Foph*, due to its specificity for cape gooseberry hosts. Here, we sequenced and assembled the genome of five strains of *F. oxysporum* from a fungal collection associated to the cape gooseberry crop (including *Foph*), focusing on the validation of the presence of SIX homologous and on the identification of putative effectors unique to *Foph*. By comparative and phylogenomic analyses based on single-copy orthologous, we found that *Foph* is closely related to *F. oxysporum* ff. spp., associated with solanaceous hosts. We confirmed the presence of highly identical homologous genomic regions between *Foph* and *Fol* that contain effector genes and identified six new putative effector genes, specific to *Foph* pathogenic strains. We also conducted a molecular characterization using this set of putative novel effectors in a panel of 36 additional stains of *F. oxysporum* including two of the four sequenced strains, from the fungal collection mentioned above. These results suggest the polyphyletic origin of *Foph* and the putative independent acquisition of new candidate effectors in different clades of related strains. The novel effector candidates identified in this genomic analysis, represent new sources involved in the interaction between *Foph* and cape gooseberry, that could be implemented to develop appropriate management strategies of the wilt disease caused by *Foph* in the cape gooseberry crop.

## Introduction

*Fusarium oxysporum* is a cosmopolitan ascomycete fungus that commonly inhabits agricultural soils. Rather than a single species, it is a species complex of non-pathogenic, plant pathogenic, and human pathogenic strains, termed the *Fusarium oxysporum* species complex (FOSC) (Di Pietro et al., 2003; Michielse and Rep, 2009; O’Donnell et al., 2009; Ma et al., 2013; Ma, 2014). Several hundred different members of the FOSC are able to penetrate plant roots, colonize xylem vessels and produce vascular wilt diseases in a broad range of host plants, including economically important crops such as banana, cotton, date palm, onion, brassicas, cucurbits, legumes and solanaceous species, such as tomato, eggplant, chili and cape gooseberry, but not grasses (Michielse and Rep, 2009). However, individual pathogenic isolates of *F. oxysporum* are highly host specific and have therefore been classified into different *formae speciales* (ff. spp.) according to the host they infect, e.g., strains that infect banana cannot infect tomato plants and vice versa (Lievens et al., 2008; Michielse and Rep, 2009; Ma, 2014). *F. oxysporum* has no known sexual stage and the mechanism for species diversification has been associated with the parasexual cycle through heterokaryon formation, which enables a mitotic genetic exchange between different nuclei (Glass et al., 2000; Di Pietro et al., 2003).

Comparative genomics of phytopathogens in the genus *Fusarium* [i.e., *F. graminearum, F. verticillioides*, and *F. oxysporum* f. sp. *lycopersici* (*Fol*)], revealed the presence of lineage specific (LS) chromosomes and chromosomal regions in *Fol* that were rich in repetitive elements and contained genes encoding known or putative effector proteins (Ma et al., 2010). Among them, 14 genes were identified that encode small proteins secreted into the xylem sap of tomato plants infected with *Fol* (called SIX proteins) (Houterman et al., 2007; Schmidt et al., 2013). Three of these *SIX* genes are avirulence genes (*Avr*), with resistance (*R*) gene counterparts identified in tomato (Simons et al., 1998; Rep et al., 2004; Houterman et al., 2008, 2009; Catanzariti et al., 2015, 2017).

Small proteins secreted by a broad range of plant pathogens, including bacteria, fungi, oomycetes and nematodes, that interfere with the cellular structure and function of their hosts are known as effector proteins (Kamoun, 2006; Kamoun et al., 2007; Hogenhout et al., 2009). The low level of homology among fungal effectors makes it difficult to identify common features that allow their classification as a group or protein family (Stergiopoulos and de Wit, 2009; de Guillen et al., 2015; Lo Presti et al., 2015). Nevertheless, many fungal effectors have been identified based on the presence of a signal peptide sequence for secretion, small size of around 300 amino acids or less, and the fact that they are often cysteine rich (Sperschneider et al., 2015). A large-scale search for putative effector genes in 59 strains of various ff. spp., resulted in a set of 104 candidate effectors including the 14 secreted in the xylem (SIX) genes, identified in *Fol* (Ma et al., 2010; Schmidt et al., 2013; van Dam et al., 2016). From this candidate effector repertoire, strains were classified according to the putative effector sequences they shared. Interestingly, all the cucurbit-infecting ff. spp. (*melonis*, *niveum*, *cucumerinum*, and *radicis-cucumerinum*), were grouped together in a separate supercluster, sharing an overlapping set of putative effectors and possibly conferring the ability to those ff. spp. to infect cucurbit host species (van Dam et al., 2016). This supercluster includes a substantial overlap with *SIX1, SIX6, SIX8, SIX9, SIX11 and SIX13* and largely excluded *SIX2, SIX3, SIX4, SIX5, SIX7, SIX10, SIX12*, and *SIX14*. Homologous of *Fol* SIX genes have been identified in alliaceous, legumes, musaceous, solanaceous, and narcissus infecting ff. spp. of *F. oxysporum* (Taylor et al., 2016, 2019; Williams et al., 2016; Czislowski et al., 2018; Simbaqueba et al., 2018).

Cape gooseberry (*Physalis peruviana*) from the Solanaceae family, is a tropical native fruit of South America found typically growing in the Andes. In Colombia, over the last three decades, the cape gooseberry has been transformed from a wild and under-utilized species to an important exotic fruit for national and international markets and represents one of the most exported fruit for Colombia (Simbaqueba et al., 2011; Moreno-Velandia et al., 2019). The cape gooseberry is also appreciated by its nutritional and medicinal properties (Yen et al., 2010; Ramadan, 2011; El-Gengaihi et al., 2013; Ramadan et al., 2015). However, despite its significant value, cape gooseberry production has been limited due to the lack of known cultivars and the absence of adequate phytosanitary measures. One of the most important disease problems in cape gooseberry is the vascular wilt disease caused by *F. oxysporum*. This disease was first described in 2005 and has become one of the limiting factors for cape gooseberry production and export (Moreno-Velandia et al., 2019). Field observations indicated typical symptoms of a vascular wilt disease with an incidence ranging from 10 to 50% with losses in production of 90% approximately (unofficially reported), in the Cundinamarca central region of Colombia. Consequently, producers moved to other places in the same region, spreading contaminated plant material and seeds (Barrero et al., 2012).

From 2012 to 2015, a total of 136 fungal isolates were obtained from cape gooseberry plants showing wilting disease symptoms, collected from different locations of the central Andean Region of Colombia. The fungal isolates were described as *F. oxysporum*, using Koch postulates and molecular markers for intergenic spacers (IGS) and the translation elongation factor 1 alpha (EF1α) gene of *F. oxysporum* (AGROSAVIA, Unpublished results). A strain of *F. oxysporum* named MAP5, was found to be highly virulent in a number of cape gooseberry plant material, including a commercial variety and different accessions from National Germplasm Bank and different collections (Enciso-Rodríguez et al., 2013; Osorio-Guarín et al., 2016). Further RNAseq analysis was performed to study differential gene expression comparing susceptible and resistant cape gooseberry plants inoculated with MAP5 (AGROSAVIA, Unpublished results). This RNAseq data was used in comparative transcriptomics, identifying eight homologous of effector genes between *Fol* and MAP5. Thus, describing a newly forma specialis of *Fusarium oxysporum* that affect cape gooseberry plants, designated as *F. oxysporum* f. sp. *physali* (*Foph*) (Simbaqueba et al., 2018).

In this study, we sequenced the genome of five fungal strains, including *Foph*_MAP5, three additional pathogenic and one non-pathogenic strains of *F. oxysporum* in cape gooseberry. We performed comparative genomics using the resulted genome assemblies to infer the phylogenetic relationship of *Foph* within the *F. oxysporum* clade. This result showed the polyphyletic origin of *Foph* and the closer relationship with ff. spp. related to *Solanaceous* hosts. We also identified putative LS genomic regions specific to virulent strains of *Foph*, that could be related with pathogenicity and host specificity, as they contain the homologous effectors previously reported for *Foph* MAP5and eight new putative effector genes identified in this study. We mapped the *Foph* RNAseq dataset previously reported against the candidate effectors and identified that these novel effectors are expressed during host infection. These results suggest that the new effector candidates, could have a putative role in virulence. Additionally, we tested the presence of the novel effectors by PCR amplification in a panel of 36 *F. oxysporum* isolates (including MAP5), associated to the cape gooseberry crop and identified that the presence of novel candidates was unique to *Foph* related strains, suggesting host specificity toward cape gooseberry plants. Furthermore, we conducted a phylogenetic analysis using the EF1alpha sequences available for this panel of *F. oxysporum* isolates. This result reflects the polyphyletic origin of *Foph* and suggests the independent acquisition of the novel putative effectors in at least two divergent clades of *Foph* related strains.

## Materials and Methods

### Fungal Material

The strains *Foph*_MAP5, *Foph*_13, *Foph*_36, Foph_72, and *Foph*_117, classified as *F. oxysporum*, were selected from a collection of 136 fungal isolates obtained from cape gooseberry crops, based on their ability to cause wilting symptoms (*Foph*_MAP5, *Foph*_13, and *Foph*_117), non-pathogenic (*Foph*_36) on susceptible cape gooseberry plants.

### DNA Extraction

The five fungal strains were reactivated in PDA media and incubated at 28°C for 8 days or until enough biomass was obtained for DNA extraction. The DNA of MAP5 strain (*Foph*), used for genome sequencing, was obtained using the ZR Fungal/Bacterial DNA kit from Zymo research^®^, according to the protocol proposed by the manufacturer. The DNA of the remaining *F. oxysporum* isolates used in this study, was extracted from 100 mg of the mycelia, using the cetyltrimethylammonium bromide (CTAB) protocol modified for fungal DNA (Zhang et al., 2010). The quality and DNA concentration using both methodologies were verified in 1% agarose gel using the 1 Kb Plus DNA Ladder (Invitrogen^®^) and also by Nanodrop DNA/RNA Quantification system.

### *Foph* Genome Sequencing and Assembly

Libraries were generated from purified DNA with the Illumina Nextera XT DNA Sample Preparation Kit (San Diego, California, United States). The resulting libraries were verified in the Bioanalyzer Agilent 2100, using a DNA-HS chip and adjusted to a final concentration of 10 nM. Libraries were then amplified. The sequencing of the libraries was performed using the TruSeq PE Cluster V2 (Illumina, San Diego, CA) kit generating 250 bp pair-end reads in the Illumina MiSeq platform (San Diego, CA, United States) at the Genetics and Antimicrobe Resistance Unit of El Bosque University.

The quality of the reads produced was verified with the software FastQC (Andrews, 2015), and reads were trimmed using the software Trimmomatic (Bolger et al., 2014), with the following parameters “LEADING:3 TRAILING:3 SLIDINGWINDOW:4:15 MINLEN:45.” Additionally, adaptor sequences and reads less than 25 bp in length were filtered and removed using the scripts fastq_quality_trimmer and fastq_quality_filter of the FASTX-toolkit platform^1^. A primary *de novo* assembly was performed with the pair-end reads overlapped into contigs, using the software, MEGAHIT v1.2.9 (Li et al., 2015), Newbler v 2.0.01.14. (454 Life Sciences), Velvet (Zerbino and Birney, 2008), and SPAdes, v 3.5.0. (Illumina, San Diego CA). The Quality Assessment Tool for Genome Assemblies (QUAST) software (Gurevich et al., 2013), was used to determine the best genome assembly based on the highest N50 parameter.

### Gene Prediction and Annotation

*Ab initio* gene models for the genome sequence of *Foph*, were predicted using the software Augustus (Stanke and Morgenstern, 2005), using the gene prediction model for *Fol*4287, as species gene model with the following parameters “–strand = both” and “–uniqueGeneId = true,” other parameters were used with the default settings. The resulted transcripts were annotated by combining predictions using the software HMMER 3.0 (Finn et al., 2011), with the PFAM protein database. The functional annotation of the transcripts was performed with the software eggNOG-mapper v4.5.1 (Huerta-Cepas et al., 2017). Gene models were corroborated with the *Foph in planta* RNAseq database reported in our previous study.

### Comparative Genomics Analysis

The comparative genomic analysis was carried out to establish the gene composition similarity and conserved patterns within phylogenetic clusters of 22 genomes of different *F. oxysporum* ff. spp. (including *Foph*), and the genome sequence of *F. fujikuroi* (Supplementary Table S1). To identify these gene clusters, we used the anvi’o software (Eren et al., 2015), following the pangenomic workflow described before (Delmont and Eren, 2018). In brief, this pipeline generates a genome database that stores DNA and amino acid sequence information of all genomes. Gene clusters were identified by calculating the similarities of each amino acid sequence in every genome against every other amino acid sequence using Blastp (Altschul et al., 1990) and finally hierarchical clustering was performed using the Euclidean distance and Ward clustering algorithm. The distribution of these gene clusters across the genomes was plotted using the anvi’o visualization tool. To reconstruct the phylogenetic relationship of these genomes, the single copy orthologous genes (SCG) were extracted from the pangenome database for all genomes, and a phylogenomic tree was generated using the FastTree 2.1 software (Price et al., 2010) as a component of the anvi’o pipeline. To root the tree, we used the genome sequence of *F. fujikoroi* as an outgroup (Supplementary Table S1).

### Identification of Effector Genes in *Foph*

To validate the presence of homologous effectors (i.e., SIX, Ave1, and FOXM_16303), identified in our previous *Foph-*MAP5 transcriptomic analysis, we carried out two search strategies of the homolog effectors in a database that included the 22 genome sequences of the ff. spp. of *F. oxysporum* used for comparative genomics of *Foph.* The first strategy consisted in a tBlastn search. The hits with an *e*-value <0.0001 and identity higher than 50%, in the 50% of the length of the sequence query, were selected for further analysis. In the second strategy, a Blastx search was performed to identify all possible putative peptides of the homologous effectors in the *F. oxysporum* genome database. The best hits with an e-value <0.0001 were selected for further analysis.

To identify *de novo* candidate effector genes in *Foph*, the secretome and effectorome were predicted from the proteome of *Foph_*MAP5, using the software SignalP v5.0 (Almagro Armenteros et al., 2019) and EffectorP v2.0 (Sperschneider et al., 2018), respectively. In order to discard homologous sequences in other ff. spp., The two BLAST search strategies mentioned above were performed using the protein sequences positive for signal peptide and effector structure (i.e., <300 aa in length and cysteine rich) as a query. An additional search of Miniature Impala Transposable Elements (*mimp*) was performed in the UTR of the transcripts predicted of *Foph*, with the regular expression “NNCAGT[GA][GA]G[GAT][TGC]GCAA[TAG]AA,” using a customized Perl script as described by Schmidt et al. (2013) and van Dam and Rep (2017), to determine whether or not the novel candidate could correspond to SIX type genes.

### Molecular Characterization of *Foph* Isolates and PCR Analysis of Candidate Effectors

A panel of 39 *F. oxysporum* isolates (including the highly virulent *Foph*_MAP5, 13, 72, 117 and the non-virulent *Foph*_36), derived from the collection fungal collection mentioned above were selected based on their ability to cause wilting symptoms on susceptible cape gooseberry plants (Supplementary Table S2). The *EF1a* gene of *Fol* (GenBank XM_018381269), was used as a molecular marker to characterize the *Foph* isolates to species level. EF1a sequences for seven out of 39 isolates (including MAP5) were obtained from the GenBank, while the EF1a sequences of *Foph*_36, 72, 117 were predicted from their genome assemblies (Supplementary Table S2). For the remaining 28 isolates, a fragment of the EF1a gene was amplified and sequenced using the primers reported by Imazaki and Kadota (2015). PCR reactions were conducted with Taq DNA Polymerase (Invitrogen^TM^, Carlsbad, CA, United States), in a 25 μL reaction volume. The PCR reaction consisted of 0.25 μL Taq Polymerase, 2.5 μL of 10X buffer (Invitrogen^TM^, Carlsbad, CA, United States), 0.16 μM of each primer, 0.16 mM of dNTP mix, 2 mM MgCl_2_ and 25 ng of template DNA. PCRs were carried out with an initial denaturing step at 95°C for 2 min followed by 30 cycles of denaturing at 95°C for 45 s, annealing of primers at 59°C (62°C for *Forl*_155.3) for 45 s and primer extension at 72°C for 45 s. The PCR was completed by a final extension at 72°C for 10 min. PCR products were purified using a QIAquick PCR Purification Kit (Qiagen) and then sequenced by Sanger platform.

EF1a sequences obtained from 27 out of the total of 39 *Foph* related isolates included in this study, were submitted to the GenBank with accession numbers (MT738937-MT738958 and MW233573-MW233575) and a total of 30 EF1a sequences of *Foph* related strains were aligned (MUSCLE method) using MEGA version 7 (Kumar et al., 2016). The corresponding EF1a sequence from the selected *F. oxysporum* ff. spp. mentioned above, were also included for comparison. Phylogenetic analysis was performed using the software BEAST (Bayesian Evolutionary Analysis Sampling Trees) v 2.6.1 (Bouckaert et al., 2019), with default settings. The resulting phylogenetic trees were visualized using the Interactive Tree of Life (iTOL) v4 (Letunic and Bork, 2019). The EF1a from *F. fujikuroi* was used as an outgroup. To corroborate the presence of the new effectors in the *Foph* related strains, specific primers for the new candidates were designed and used for PCR amplification (Supplementary Table S3), using the same conditions as mentioned above. DNA from Colombian strains of *Fol, Foc* R1 and TR4, were provided by Dr. Mauricio Soto (AGROSAVIA), and used as a control for amplification.

## Results

### *Foph* Genome Sequencing and Assembly

The genome sequence of four pathogenic strains of *Foph* (i.e., MAP5, 13, 72, and 117 and one non-virulent named *Foph*_36 in cape gooseberry plants, were assembled from 250 bp paired end reads Illumina MiSeq. The genome of *Foph*_MAP5 was assembled into 1,856 contigs with a total size of 44.9 Mb. This genome assembly is smaller, compared to the remaining assemblies of *Foph* strains obtained in this study ∼46–48 Mb, other Illumina genome assemblies available for *Fophy* strains (infecting a close related host *Physalis philadelphica*, known as “husk tomato or tomatillo”), two solanaceous specific strains (Fomel_002 and Fonic_003), and the nearly complete genome assemblies of *Fol*4287 and FoC_Fus2, included here as reference genomes (Table 1; Ma et al., 2010; van Dam et al., 2016, 2017a; Armitage et al., 2018). The difference in size and contig number in the assembly of *Foph*_MAP5, might be a consequence of genome fragmentation due to the sequencing based on short reads (250bp) used in this study. Nevertheless, the predicted gene content of *Foph*_MAP5 (14,897 transcripts), is similar to other Illumina genome assemblies of different ff. spp. of *F. oxysporum*, available in the GenBank (Table 1 and Supplementary Table S1).

**TABLE 1:** *Foph* genome assembly statistics, compared to other Illumina genome sequences of *F. oxysporum* strains infecting *Solanaceous* hosts and two nearly complete genome assemblies of *Fol* and *FoC*.

**Strain**	**No. of contigs**	**Maximum length (kb)**	**N50 (kb)**	**GC (%)**	**Assembly length (Mb)**	**Transcripts**
*Foph* MAP5	1,856	453	70	48.5	44.9	14,897
*Foph 13*	1,395	547	125	47.6	46.2	14,736
*Foph 36*	1,701	547	117	47.5	48.2	15,379
*Foph 72*	1,646	898	126	47.7	48.2	15,389
*Foph 117*	1,417	492	1,191	47.6	46.4	14,775
*Foph*y KOD886	488	2,037	1167	47.7	47.2	23,095
*Foph*y KOD887	1,275	1,667	547	47.6	50.4	24,279
Fomel 001	1,725	2,348	227	47.5	52.3	16,492
Fonic 003	638	2,572	1159	47.6	49.9	15,480
*Fol*_4287*	88	6,854	458	48.3	61.4	27,347
FoC_Fus2*	34	6,434	414	47.7	53.4	19,342

*^∗^Genome assemblies obtained from PacBio sequencing technology.*

### Comparative Genomics of *Foph* With Other Isolates of *Fusarium oxysporum*

**FIGURE 1:**
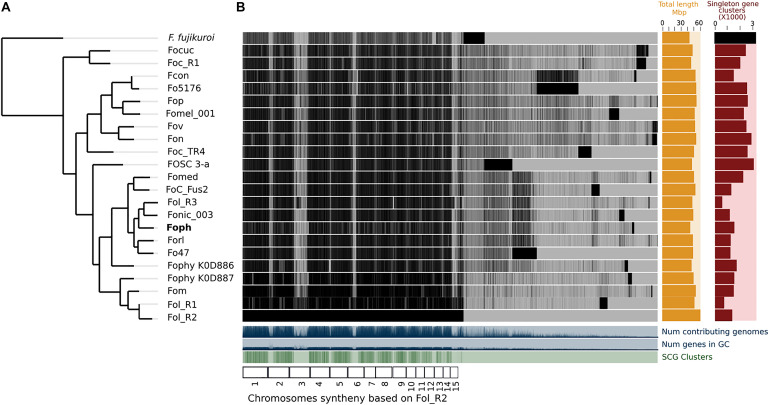
Comparative genomics between *Foph* and 21 *F. oxysporum* ff. spp. **(A)** The phylogenomic tree was inferred with the single copy orthologous genes of the 22 genomes of *F. oxysporum* used in this analysis. The amino acid sequence of the translated genes was concatenated, and the final alignment consists of a total of 41,84,361 amino acid positions. The phylogenetic tree was constructed using FastTree 2.1. *F. fujikoroi* (IMI58289), was used as outgroup. **(B)** Pangenomic analysis of *F. oxysporum*, showing the core genome the species complex and single copy orthologous genes, possibly forming the LS genome for each forma specialis. The *F. oxysporum* pan-genome was generated using the anvi’o pangenomic workflow.

A total of 14,897 transcripts were predicted from the genome assembly of *Foph*_MAP5 (used here as a reference of *Foph* pathogenic strains), from which 14,140 have an orthologous counterpart in the genomes of *F. oxysporum* compared in this study (Table 1). Using the anvi’o pipeline for pangenome analysis, a set of the single copy orthologous genes (SCG) present in the 22 *F. oxysporum* genomes were extracted to reconstruct their phylogenetic relationship. We used this phylogenetic reconstruction to test whether *Foph* could be related to *Fophy* (i.e., other *Physalis* infecting strains), or might be grouped in a lineage of strains that infect *Solanaceous* hosts. The phylogenomic tree showed that *Foph* shared the same clade with *Fonic* and *Fol_*R3 and is closely related to *Forl*, *Fo47* (both strains associated to the tomato crop) and more distantly related to *Fophy*_KOD886, while the remaining solanaceous infecting strains are grouped on a different clade (Figure 1A), reflecting the polyphyletic origin of infecting strains of *F. oxysporum* and their host specificity. We also performed a comparative analysis using the SCG shared between *Foph* and the remaining 21 genomes of *F. oxysporum* ff. spp., This analysis showed that the majority of *Foph* SCG (∼14 K), are syntenic with the core chromosomes of *Fol* (used here as the reference genome sequence of *F. oxysporum* species). The syntenic SCG might correspond to the core genome of *Foph*, while the remaining ∼0.5 K of *Foph* SCG, could correspond to transcripts that are not present in any cluster and their contigs could be part of the LS genomic regions specific of *Foph* (Figure 1B).

### Effector Homologous Are Confirmed in *Foph* Pathogenic Strains

**FIGURE 2:**
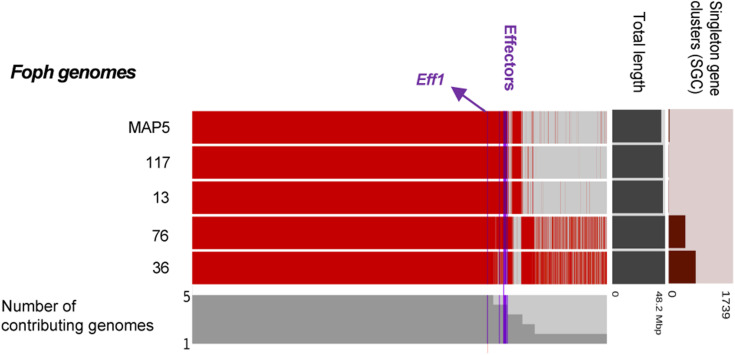
Comparative genomic analysis of *Foph* isolates. The comparative genomic analysis of four pathogenic strains, *Foph_*MAP5, *Foph_*13, *Foph*_72, and *Foph_*117, and one non-pathogenic strain, *Foph* 36, was conducted to establish the possible lineage-specific regions based on the patterns of gene sharing. The cluster of shared and unique genes are shown by the red lines. Gene clusters are organized by their presence in all five genomes to those present in only one genome, indicated by the gray bars. The horizontal bar graphs to the right represent the total length of the genome and the number of singleton gene clusters for each genome. Blue vertical lines show the location of the SIX effectors as well as the putative novel effectors. The majority of putative and known effectors are only present in the gnomes of pathogenic strains, while effector *Eff1* is present in all Foph genome assemblies, including the non-pathogenic *Foph_*36.

In our previous study, eight homologous effectors were identified in *Foph* by *in planta* RNAseq mapping analysis with the LS regions of *Fol*. Here, we performed a combination of Blastp and Blastx searches of the known SIX effectors and Ave1 effectors in the genome assemblies of the five strains of *Foph* and the genome sequences of 21 *F. oxysporum* ff. spp., used for comparative genomics (Supplementary Table S1). This result showed the widespread presence of SIX homologous in different ff. spp. of *F. oxysporum* and confirmed the presence of highly identical *Fol* and *Fomed* homologous effectors with identities from 87 to 100% in their counterparts of *Foph* virulent strains MAP5, 13, 72, and 117 and absent in the non-pathogenic strain *Foph*_36 (Table 2). Interestingly, we compared the genome assemblies of *Foph* strains using the anvi′o software and showed that the putative effectors are located in genomic regions unique to *Foph* virulent strains (Figure 2), which could correspond to the LS regions of *Foph*. Furthermore, we also identified a highly identical putative homologous transcript of the *Fol* SIX13 effector in the genomes *Foph* virulent strains. This prediction was manually confirmed as the corresponding transcript of SIX13, was fragmented into two contigs (ctg_1292 and ctg_1535) in the genome of *Foph*_MAP5 (Table 3). We used the *Foph*_MAP5 prediction of the SIX13 transcript to search the corresponding homologous sequence in the remaining genome sequences of *Foph* generated in this study and confirmed the presence of SIX13 homolog only in the *Foph* virulent strains (Figure 2 and Table 2).

**TABLE 2:** tBlastn identities of *Foph* effectors compared against the *F. oxysporum* species complex WGS databases.

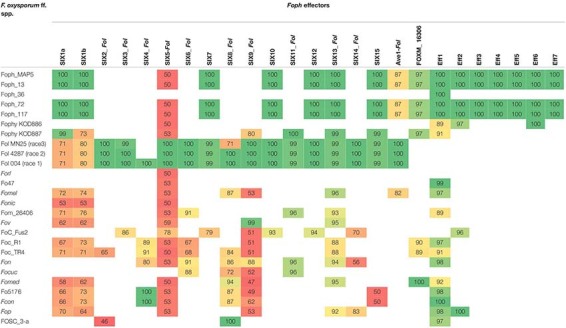

*Coloured cells indicate aminoacidic identities Dark green = Highly identical proteins (100%) to red = somehow similar proteins (∼50%).*

**TABLE 3:** Genomic analysis of the effectors identified in *Foph_*MAP5.

**Genomic features**		***Foph* effectors**															
		**Homologous**									**Novel candidates**						
		**SIX1a**	**SIX1b**	**SIX7**	**SIX12**	**SIX10**	**SIX13**	**SIX15**	**Ave1**	**FOXM_16306**	**eff1**	**eff2**	**eff3**	**eff4**	**eff5**	**eff6**	**eff7**
Gene	Contig	593	569	568		789	1,292–1,535	709	1,018	1,149	583	692	1,304		1,453	1,487	359
	Contig size (kb)	7.9	5.4	15.86		4.8	1.6–1	0.8	2.7	1.2	4.3	4.8	2.1		1.3	0.3	22.5
	Length (bp)	874	855	491	432	520	941	403	378	366	493	491	519	604	343	384	252
	CDS (bp)	874	855	491	384	450	774	300	378	366	267	270	519	456	291	384	252
	*mimp* class	4	NM	1	1, 2		NM	NM	2	1	4	NM	NM	1	NM	NM	NM
Protein	Length (aa)	285	284	163	128	150	258	100	125	122	89	90	173	151	93	127	84
	SignalP	Y	Y	Y	Y	Y	Y	Y	Y	Y	Y	Y	Y	Y	Y	Y	Y
	TMHMM	0	0	0	0	0	0	0	0	0	0	1	0	0	0	0	0
	EffectorP	Y	Y	Y	Y	Y	N	Y	Y	Y	Y	Y	Y	Y	Y	Y	Y
	ApoplastP	N	N	Y	Y	Y	N	N	Y	Y	Y	Y	Y	Y	Y	N	Y
RNAseq reads aligned		4.1	10.1	3	4.7	3.4	0	3.4	2.5	2.1	20	7	22	2	14	5	0

*NM, no mimp element identified*.

**FIGURE 3:**
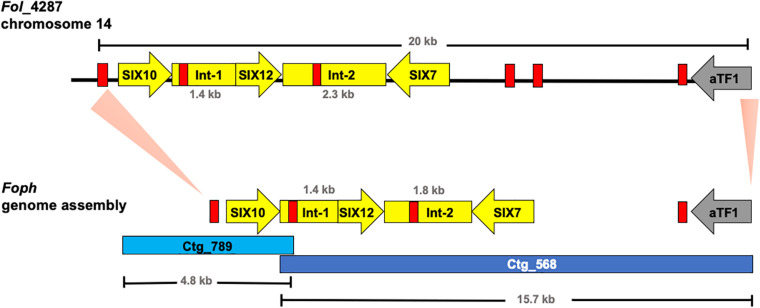
Graphical representation of a 20 kb segment of the chromosome 14 in *Fol*, containing the cluster of effectors SIX10, 12 and 7, and the aTF1 gene (FOXM_17458) (upper part). The chromosomal segment is conserved in the *Foph* genomic region (shown by orange pale triangles), corresponding to the overlapped Contigs 568 and 789 (Bottom part), suggesting a highly possible horizontal transfer of a chromosomal segment of 20 kb between both ff. spp. Int-1 = conserved intergenic region between SIX10 and SIX12. Int-2 = conserved intergenic region between SIX12 and SIX7. Red blocks represent *Mimp* transposable elements flanking the cluster of effector genes shared between *Fol* and *Foph.*

These results also confirmed that the *Fol* effector gene cluster formed by the SIX7, 12 and 10 (Ma et al., 2010; Schmidt et al., 2013) and partially identified in *Foph* by *in planta* transcriptomics (Simbaqueba et al., 2018), is entirely conserved in the genome of *Foph.* SIX7 and SIX12 homologous are both present in the same contig (ctg_568) while SIX10 is located in another contig (ctg_789) of the *Foph*_MAP5 genome assembly (Table 3). Thus, we manually inspected the sequences of these contigs and found that both contigs are overlapped by a sequence segment of 22 bp at the proximal 5′ end of the ctg_586 with the distal 3′ end of the ctg_789. This overlapped segment of both contigs correspond to a *mimp* class 2 sequence in intergenic region between SIX10 and SIX12. The *Foph* effector gene cluster is 4.7 kb in length and is similar to that formed by the same homologous effectors in *Fol* (5.2 kb), including the intergenic regions with the approximate same length as *Fol* (1.8 kb between SIX7 and SIX12 and 1.4 kb between SIX12 and SIX10, respectively), and three *mimp* elements that flank the effector gene cluster reported by Schmidt et al. (2013) in *Fol* (Figure 3).

Further inspection of the ctg_568 in *Foph*_MAP5, also confirmed the presence of another highly conserved homologous gene (FOXG_17458) between *Foph* and *Fol*, including the corresponding *mimp* class 1 element in the 5′ UTR (Figure 3). The transcript FOXG_17458 in *Fol*, encode a transcription factor of the family *a*TF1—FTF1 (van der Does et al., 2016), and is located 9 kb away from the ORF of the SIX7 (Schmidt et al., 2013). Intriguingly its homologous counterpart presented in the *Foph* genome is located 7 kb away from the SIX7 ORF (Figure 3). Although, there is a difference of 2 kb that includes two *mimp* elements presented in *Fol*, comparing the above-mentioned intergenic regions in both genomes. This finding points out to a probable horizontal transfer event of a chromosomal segment of at least 20 kb in length between *Fol* and *Foph*. In *Fol*, SIX15 is a non-annotated transcript and is located 55 kb away from the *a*TF1. This chromosome region includes other four annotated transcripts: FOXG_17459, FOXG_17460, FOXG_17461, and FOXG_17462. Thus, we performed a Blastn search using this sequence of 55 kb from *Fol* as a query and compared with the *Foph*-MAP5 genome assembly, in order to test whether an extended sequence of the chromosome 14 of *Fol* might be conserved in *Foph.* However, no additional chromosomal segment shared between *Fol* and *Foph* was identified by comparing both genomic sequences.

### Novel Candidates for Effector Genes in *Foph*

We identified novel effector genes in the *Foph*_MAP5 genome, by combining the sets of proteins from the secretome and effectorome. We predicted a total of 1,495 secreted proteins, forming the secretome of *Foph*_MAP5, from which 276 were determined to be effectors, named herein as “*Foph* effectorome.” Six transcripts of the *Foph* effectorome (named *Eff*2 to 7), were identified as putative novel effectors, due to their specificity to *Foph* virulent strains and their lack (i.e., *Eff2*, *Eff3*, *Eff4*, and *Eff7*) or low similarity (*Eff5* and *Eff6*) to any protein reported in the public databases (Table 2). Additionally, *mimp* elements were identified 624and 430 bp upstream from the transcripts *Foph_eff2* and *Foph_eff5*, respectively (Table 3).

**FIGURE 4:**
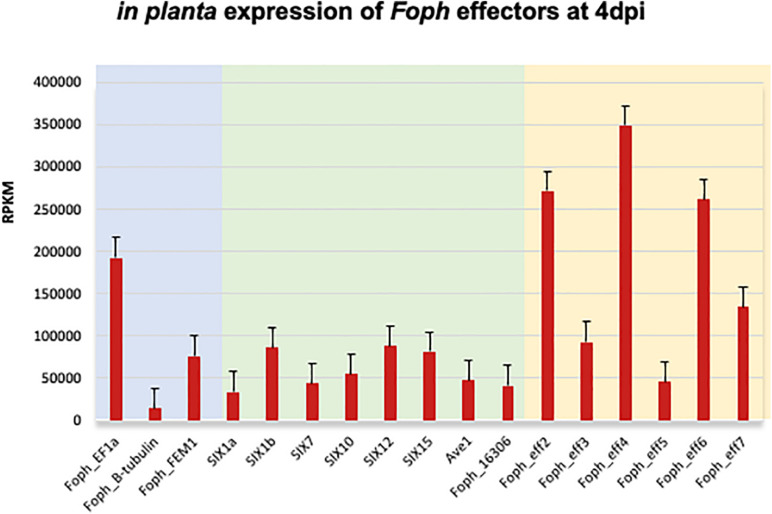
Expression analysis of the effectors identified in the genome sequence of *Foph_*MAP5, using the RNAseq data form cape gooseberry susceptible plants inoculated with *Foph* at 4 dpi, reported in our previous *in planta* transcriptomic analysis. Pale blue panel indicate the genes translation elongation factor alpha (EF1a), tubulin B-chain (B-tubulin), and *Fusarium* Extracellular Matrix 1 (FEM1), to use as constitutive expressed control genes of *Foph* during host infection. Pale green indicates the expression of the homologous effectors identified in *Foph.* Pale yellow indicates the expression of the newly identified effectors in *Foph*. Six out of seven new effector candidates are expressed during cape gooseberry infection with a higher expression of *eff2*, *eff4*, and *eff6*, compared to the rest of the effectors analyzed. RPKM, reads per kilobase per million of mapped reads. Scale bars indicate standard error.

The candidate effector *Eff1*, was the only predicted putative effector present in all the sequenced strains, including the non-pathogenic Foph_36 (Figure 2). In addition, *Eff1* showed significant tBLASTn hits with different *F. oxysporum* ff. spp., including another non-pathogenic strain Fo47. Therefore, this transcript could be excluded as a novel effector gene. The putative effectors *Eff3* and *Eff4* are clustered in the contig_692 at 700 bp of distance approximately between them in the genome of *Foph*_MAP5. Furthermore, we predicted a transmembrane domain for protein encoded by *Eff3* (Table 3), suggesting a cellular localization and with a possible different function from a secreted protein. Additionally, we performed an RNAseq mapping against the ORF of the novel candidate effectors and found that all six putative effectors are expressed in *Foph*_MAP5 during cape gooseberry infection at 4 dpi. In this analysis, we also included the homologous of SIX effectors and the homologous transcripts of EF1alpha, β*-tubulin* and *Fusarium* extracellular matrix 1 (*FEM1*) in *Foph*_MAP5, as housekeeping genes for expression controls. We found that *Eff2*, *Eff4, Eff6*, and *Eff7*, showed higher expression compared to the rest of the transcripts analyzed (Figure 4). Interestingly, *Eff2, Eff4*, and *Eff6*, showed higher expression, compared to all three-housekeeping gene controls. This *in planta* expression evidence of the putative novel effectors, together with their specific presence in the genomes of virulent strains of *Foph*, suggest that these genes could be involved in *Foph* pathogenicity toward cape gooseberry hosts.

### Novel Effectors Are Present in *F. oxysporum* Isolates Associated to the Cape Gooseberry Crop

**FIGURE 5:**
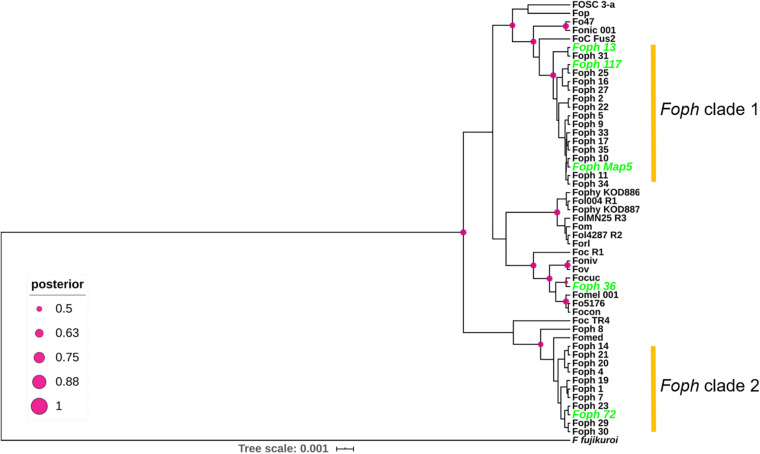
Phylogenetic tree of a partial sequence of the EF1a gene from the genome sequences of 24 ff. spp. of *F. oxysporum* (Supplementary Table S1) and 29 *F. oxysporum* isolates obtained from cape gooseberry crops (Supplementary Table S2). The phylogenetic analysis was conducted using BEAST. Node shapes indicate the bootstrapping support, indicated as Bayesian posterior probabilities. The scale bar indicates time in millions of years.

In order to test whether the candidate effectors genes could be used as potential molecular makers for *Foph* identification in diagnostic strategies, we performed a preliminary screening of the novel candidate effectors by PCR amplification in a panel of 37 *F. oxysporum* isolates (including *Foph-*MAP5), obtained from cape gooseberry crops. Thirty-three of them (including the sequenced strains MAP5 and 13), have been classified as pathogenic due to their ability to cause wilting symptoms on a susceptible cape gooseberry genotype, while the remaining four isolates (including the sequenced strain *Foph*_36), do not cause wilting symptoms on cape gooseberry plants. Thereby, classified as non-pathogenic strains of *Foph* (Supplementary Table S2). The screening also included DNA isolated from *Fol*, *Foc*R1, and *Foc*TR4 strains, as control for amplification. We found amplification for all candidates in the majority of *F. oxysporum* isolates associated with cape gooseberry, including pathogenic and non-pathogenic isolates (Supplementary Table S2 and Supplementary Figure S1). The putative novel effector *Eff*2 was identified by PCR amplification in *Foph*_36 (Supplementary Figure S1), but was absent in our genome analysis (Figure 2 and Table 2), contradicting the molecular identification. Although the genomic evidence of the five sequenced strains *Foph* suggest that the putative novel effectors could be specific to virulent strains of *Foph*, the PCR screening in the panel of *Foph* pathogenic and non-pathogenic isolates, showed a more widespread presence of these putative effectors (Supplementary Table S2). Interestingly, we did not identify the presence of the novel effectors *Eff*3 to 7 in the control strains *Fol*, *Foc*R1 and *Foc*TR4. Nevertheless, together, these results, would suggest that five of the six putative novel effectors could be specific for *F. oxysporum* strains associated to the cape gooseberry crop. We also conducted a molecular characterization using the *EF1alpha* sequence in 30 out of 37 *Foph* and the additional sequenced pathogenic strains *Foph* 72 and 117, in order to test whether these isolates associated to cape gooseberry plants, might originated from a single linage. The phylogenetic tree showed that *Foph* isolates are grouped together in two different lineages, suggesting their polyphyletic origin. Additionally, we found that the pathogenic strains MAP5, 117 and 13 are grouped together in the clade 1 and intriguingly, the non-pathogenic strain *Foph*_36 was grouped on a different clade out of *Foph* (Figure 5).

## Discussion

### *Foph* Genome and Phylogenetic Relationship With Other ff. spp.

In Colombia, the cape gooseberry crop is severely affected by pathogenic strains of *Foph*, with losses of nearly 90%. In this pathosystem, SNPs associated to resistant cape gooseberry genotypes, *Foph* pathogenic strains and homologous effectors have been identified (Osorio-Guarín et al., 2016; Simbaqueba et al., 2018). However, there is a need to implement genomic approaches to corroborate these findings and to identify new sources associated to the interaction between *Foph* and cape gooseberry. These approaches could be used in the development of disease management strategies and plant breeding programs in the cape gooseberry crop. Here we sequenced and assembled the genomes of four virulent strains of *Foph* (MAP5, 13, 72, and 117) and one non-virulent strain (*Foph*_36), aiming to identify novel candidates for effector genes unique to virulent strains, that could be characterized in further studies and implemented in diagnostic strategies. Comparative and functional genomics of *F. oxysporum* that infect cucurbit species, suggested that their host range could be determined by the close phylogenetic relationship associated to their homolog effector gene content (van Dam et al., 2016, 2017b). This hypothesis is supported by additional evidence on the formae speciales *radicis-cucumerinum* (*Forc*) and *melonis* (*Fom*), showing that a syntenic LS chromosome region is highly related to the expansion formae speciales range (van Dam et al., 2017b; Li et al., 2020a). Recent genome analysis with a chromosome-scale assembly of the brassicas infecting f. sp. Fo5176, showed a similar pattern of phylogenetic relationship possibly associated to the expansion of their host range (Fokkens et al., 2020). Here, we performed comparative genomics using the *Foph_*MAP5 genome assembly in order to test whether a set of available genomes of Solanaceous-infecting formae speciales including *Foph*, could show a similar phylogenetic related pattern. Nevertheless, our analysis showed that the tested strains have a different ancestry (Figures 1A, 5), despite the close phylogenetic relationship of *Foph* with tomato infecting ff. spp. *Fol*-R3, Fo47, *Forl*, and tobacco Fonic_003. Resequencing of the genomes including *Foph*, *Fophy*, *Fonic*, and *Fomel*, using long reads, will help to gain a deeper understanding of the phylogenetic relationship among Solanaceous-infecting ff. spp.

### Confirmation of Homologous and Identification of New Putative Effectors

Homologous of *Fol SIX* genes have been identified in other ff. spp. of *F. oxysporum* and other *Fusarium* species (Meldrum et al., 2012; Thatcher et al., 2012; Li et al., 2016; Rocha et al., 2016; Schmidt et al., 2016; Taylor et al., 2016, 2019; van Dam et al., 2016, 2017a; Williams et al., 2016; Armitage et al., 2018; Simbaqueba et al., 2018). The presence of the SIX homologous might be a consequence of horizontal transfer of genes or segments of pathogenicity chromosomes between different strains of *F. oxysporum* and/or fungal phytopathogenic species. In our previous study, we identified homologous of the SIX, Ave1 and FOXM_16306 effectors, analyzing an in *planta* RNAseq of *Foph*. Despite the fragmentation of this genome assembly (i.e., no scaffolds/chromosomes scale), we corroborated the presence of complete sequences of the homologous effectors SIX, Ave1 and FOXM_16306, in the genome sequences of *Foph* virulent strains, contained in different contigs that could correspond to the LS genomic regions of *Foph* (Figure 2 and Tables 2, 3).

We also found a homolog transcript of the *Fol* SIX13 in the genome of *Foph_*MAP5, fragmented into two contigs and corroborated in the other *Foph* sequenced genomes of virulent strains. In *Foph*_MAP5, this homolog was not expressed at 4 dpi and therefore, it was not identified in our previous transcriptomics study. SIX13 homologous are present in legume, cucurbits, musaceous and solanaceous infecting ff. spp. of *F. oxysporum* (van Dam et al., 2016; Williams et al., 2016; Czislowski et al., 2018). The later mentioned ff. spp., are highly identical at the protein level (96% in *Fomel* and 99% in *Foph* and *Fophy*, respectively) (Table 2). In cucurbits infecting ff. spp. of *F. oxysporum*, a suit of effectors was found to be associated with host specificity (van Dam et al., 2016). Thus, the highly identical SIX13 homologous in the Solanaceous-infecting ff. spp., could be related to their specificity for these group of host species. Moreover, the majority of the SIX genes in *Fol* are located on the chromosome 14 (i.e., pathogenicity chromosome), except for SIX13, which is found in the LS chromosome 6 (Schmidt et al., 2013). Similarly, SIX13 corresponding homologous of *Fomed* and *Foph* are located on putative LS regions (Williams et al., 2016; Figure 2). In *Foc*, SIX13 homologous, have been associated to the differentiation of TR4 and R4 and are currently used in molecular based diagnostic of TR4 in banana crops (Carvalhais et al., 2019). Together, this evidence suggests that SIX13 could play a role in pathogenicity or host specificity. Future functional analysis of on *Foph*-SIX13 is necessary to confirm this hypothesis.

Furthermore, we performed a manual inspection of the contigs 568 and 789 of the *Foph*_MAP5 genome and confirmed the presence of a highly conserved chromosomal segment of 20kb of *Fol* that includes a cluster of physically linked effector genes (SIX7, SIX10, SIX12, and extended transcription factor αTF1). This shared region also included their corresponding flanking *mimp* elements (Figure 3; Schmidt et al., 2013; Simbaqueba et al., 2018). This finding suggests a highly probable horizontal acquisition of an entire genomic segment of 20kb from an ancestor of *Fol* or *Foph*. Miniature impala (*mimp*) transposable elements (TEs), have been identified in the genome sequences of different phytopathogenic fungi of the *Fusarium* genus (Schmidt et al., 2013; van Dam and Rep, 2017). In *F. oxysporum*, *mimp* elements have been associated to the gain or loss of effector genes, presumably acting as an evolutionary mechanism of emergence of new phytopathogenic strains (van Dam et al., 2017b). The presence highly identical *mimp* elements, flanking the homologous effector gene cluster in both *Fol* and *Foph* (Figure 3), suggests that these TEs could play a role in the lateral transference of this homolog genomic region between *Foph* and *Fol*.

Functional analysis of SIX effectors in *Fol*, showed that mutant strains with a large deletion (0.9 Mb) of chromosome 14, including the candidate effector genes SIX6, SIX9, and SIX11 did not show any loss of virulence compared to wild type *Fol* on tomato plants (Vlaardingerbroek et al., 2016). Recent evidence revealed by another set of *Fol* mutant strains with chromosomal deletions that include the SIX10, SIX12, and SIX7 gene cluster, showed no loss of virulence on tomato plants (Li et al., 2020a). These findings indicate that the genes located in these chromosomal segments (including the SIX genes with homologous in *Foph*), could be dispensable for pathogenicity, while the remaining segments could be sufficient for tomato infection (Vlaardingerbroek et al., 2016; Li et al., 2020b). Although neither of the SIX7, SIX10, and SIX12 effector genes have a role in *Fol* virulence, the presence of the highly identical homologous between *Fol* and *Foph*, suggests that this segment could be undergoing adaptation to another environment (i.e., different host plant). Therefore, it might be possible that SIX7, SIX10, and SIX12 have a role in *Foph* pathogenicity. Future investigation about the function of this conserved genomic region between these two Solanaceous-infecting ff. spp., is required. Crossed pathogenicity assays inoculating tomato and cape gooseberry with *Fol* and *Foph* and knock out of the gene cluster in *Foph* could be performed to support these hypotheses.

In this study, we confirmed that homologous of Ave1 have been only identified in the solanaceous infecting ff. spp. *Fol, Foph, Fomel*001 (Table 2) and in the f. sp. *gladioli* of *F. oxysporum* (Simbaqueba et al., 2018). Ave1 could also be present in putative conditional dispensable segments on the *Foph* genome (Table 2 and Figures 1B, 2). The presence of less conserved homologous of *Fol* including SIX1 and Ave1, which are also located on *Fol* chromosome 14 (Schmidt et al., 2013), suggests that these effectors may have a different ancestry, via acquisition of different segments of the pathogenicity chromosome at different times in the evolution of *Fol* or *Foph*.

In the tomato pathogen *Verticillium dahliae, Ave1* is involved in pathogenicity, while there is no evidence that its homolog present in *Fol* has a role in virulence (de Jonge et al., 2012; Schmidt et al., 2013). Furthermore, *Fol-*Ave1 is not expressed during tomato infection (Catanzariti, personal communication). Conversely, we found that *Foph-*Ave1 was expressed during cape gooseberry infection (Figure 4). This finding suggests that *Ave1* might have a role in *Foph* pathogenicity. Therefore, functional analyses are required by generating gene knockout strains in *Foph.* In both *V. dahliae* and *Fol, Ave1* could act as avirulence factors since they are recognized by the tomato receptor Ve1 (de Jonge et al., 2012). The *Ave1* homolog of *Foph* is highly similar at the protein level to its counterparts in *F. oxysporum* (*Fol*, *Fomel*, and *Fogla*), and less similar to *V. dahliae* Ave1 (Table 2; Simbaqueba et al., 2018). The presence of Ave1 in *Foph*, suggests that the avirulence function of *Fol* Ave1 might be conserved. This hypothesis needs further investigation e.g., by testing for recognition of *Foph* Ave1 by tomato *Ve1* or a homolog in cape gooseberry.

Novel candidate effectors in *F. oxysporum* have been reported for other ff. spp., including *Fom*, *Foc_*Fus2, *Fonar* and legume infecting strains (Schmidt et al., 2016; Taylor et al., 2016, 2019; Williams et al., 2016; van Dam et al., 2017a; Armitage et al., 2018), based on the analysis of their genome sequences to identify transcripts that encode for small proteins with a secretion signal peptide and the proximity of mimp to the start codon. Here, we used the predicted transcripts from the genome assembly of *Foph*_MAP5 to identify novel effectors, based on the effectorome andsecretome repertoires, and the absence or low similarity to any predicted or non-predicted protein sequences compared to *F. oxysporum* genomes available in the public databases and additional genome assemblies generated in this study of four different *Foph* strains (Figure 2 and Tables 2, 3). Three highly expressed novel effectors during infection (*Foph*_*eff2*, *eff4* and *eff7*), are unique *Foph* candidate effectors, while the other highly expressed candidate *Foph_eff6*, have identical homologous proteins in the genomes of *Fomel* and *FoC*_Fus2 (Table 2). Furthermore, the homologous counterpart identified in *FoC_*Fus2 is located in a lineage specific region (Armitage et al., 2018). These findings suggest that *Foph_eff6* and its homologous, may have a putative role in pathogenicity and represent a subject for future functional analysis.

### Presence of Effectors in *Foph* Compared to Other ff. spp. of *F. oxysporum*

*Foph* pathogenic strains are responsible for the wilting disease that affect cape gooseberry crops in Colombia. Thus, appropriate disease management strategies are needed to be implemented (Barrero et al., 2012). However, the development of those strategies has been largely limited due to the lack of knowledge of the wilting disease caused by *Foph*, and accurate identification of pathogenic strains. Detection methods based on the use of effector genes as molecular markers are highly desirable for precise identification of pathogenic strains in disease management programs of soilborne pathogens due to their limited sequence diversity between members of the same *f. sp.* (Rocha et al., 2016; Gordon, 2017), thus providing a solid and sensitive identification of pathogenic strains of soilborne pathogens including *F. oxysporum* (van Dam et al., 2017a; Carvalhais et al., 2019; Taylor et al., 2019).

Comparative genomics have been performed to design molecular markers based on candidate effector genes and successfully tested for the identification of cucurbit and *Narcissus* Infecting ff. spp. of *F. oxysporum* (van Dam et al., 2016; Taylor et al., 2019). In this study, we used the highly conserved novel candidate effectors found by comparative genomics in *Foph*, to explore their usefulness as potential molecular markers specific for pathogenic strains. The presence of homologous effectors suggests a functional redundancy between different ff. spp. (Taylor et al., 2019). Here, we identified that the candidate novel effector *Foph_eff1* has homologous in other ff. spp. (Table 2). We also identified the presence of *Eff1* in all tested strains, including the non-pathogenic strain sequenced *Foph_*36 (Figure 2), *Fol* and *Foc.* Thus, the role of *Eff1* in pathogenicity may be dispensable due to its presence in different *F. oxysporum* strains and could be discarded for diagnostic purposes. The remaining novel effectors showed a clear pattern of amplification in *F. oxysporum* strains associated to the cape gooseberry crop, compared to the highly pathogenic *Fol* and *Foc* in tomato and banana respectively (Supplementary Figure S1 and Supplementary Table S2). Although the novel effectors we predicted were only identified in the genome sequences of *Foph* virulent strains (Figure 2), we did not find an amplification pattern associated in three out of the e four non-pathogenic strains compared in this analysis. A similar inconsistent pattern of presence/absence between pathogenic and non-pathogenic cucurbit infecting strains of *F. oxysporum* was observed for some of the effectors-based markers developed by van Dam et al. (2017a). These results might be supported by the fact that effectors show limited sequence diversity between strains of the same f. sp. (van Dam et al., 2017a; Taylor et al., 2019). An alternative explanation could be related to the limited number of effectors-based markers identified in this fragmented genome assembly of *Foph*-MAP5. New markers associated to *Foph* pathogenicity will be predicted in future studies, enlarging effectorome repertoire by resequencing of the *Foph* genomes with long reads sequencing technologies as performed for *Forc* and *FoC_Fus2* (van Dam et al., 2017b; Armitage et al., 2018).

## Data Availability Statement

The data presented in the study are deposited in the NCBI repository under the BioProject accession number PRJNA640423. GenBank accession numbers: MT738929 –*Foph*_MAP5 SIX13, MT38930-MT38936-*Foph*_MAP5 eff1 to *eff7*, MT738937, MW160862-MW160910 *Foph*_01, 04, 72 and 117 –*eff1* to *eff7* and homologues of SIX effectors – MT738958, MW233573- MW233575, EF1a sequences of *F. oxysporum* strains associated to cape gooseberry crops. “Access to these sequences must be requested to the Ministry of Environment and Development of Colombia. *Foph* strains used in this work were collected under the framework collection permit No.1466 from 2014 of AGROSAVIA and registered in the National Collections Registry (RNC129) of Colombia.”

## Author Contributions

JS planned and carried out the *Foph* genome analysis, planned the experiments, analyzed the data, created figures, and drafted, wrote and edited the manuscript. ER and DB-D carried out the experiments with *Foph* isolates. CG obtained funding, planned experiments, contributed and edited the manuscript. AC-Q obtained funding, planned and carried out the *Foph* genome sequencing, analysis and all bioinformatics, created figures, drafted and edited the manuscript. All authors contributed to the article and approved the submitted version.

## Conflict of Interest

The authors declare that the research was conducted in the absence of any commercial or financial relationships that could be construed as a potential conflict of interest.

## Footnotes

1 hannonlab.cshl.edu/fastx_toolkit
