# Supplementary material for: Putative Novel Effector Genes Revealed by the Genomic Analysis of the Phytopathogenic Fungus Fusarium oxysporum f. sp. physali (Foph) That Infects Cape Gooseberry Plants
Source: Front Microbiol. 2021 Jan 18;11:593915. doi: 10.3389/fmicb.2020.593915 (PMC7847934; doi:10.3389/fmicb.2020.593915)
Supplement: Supplementary file 4 [file Image_1.pdf]

A.

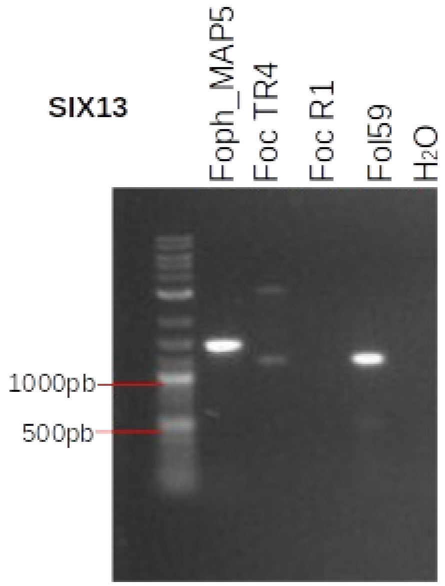

B.

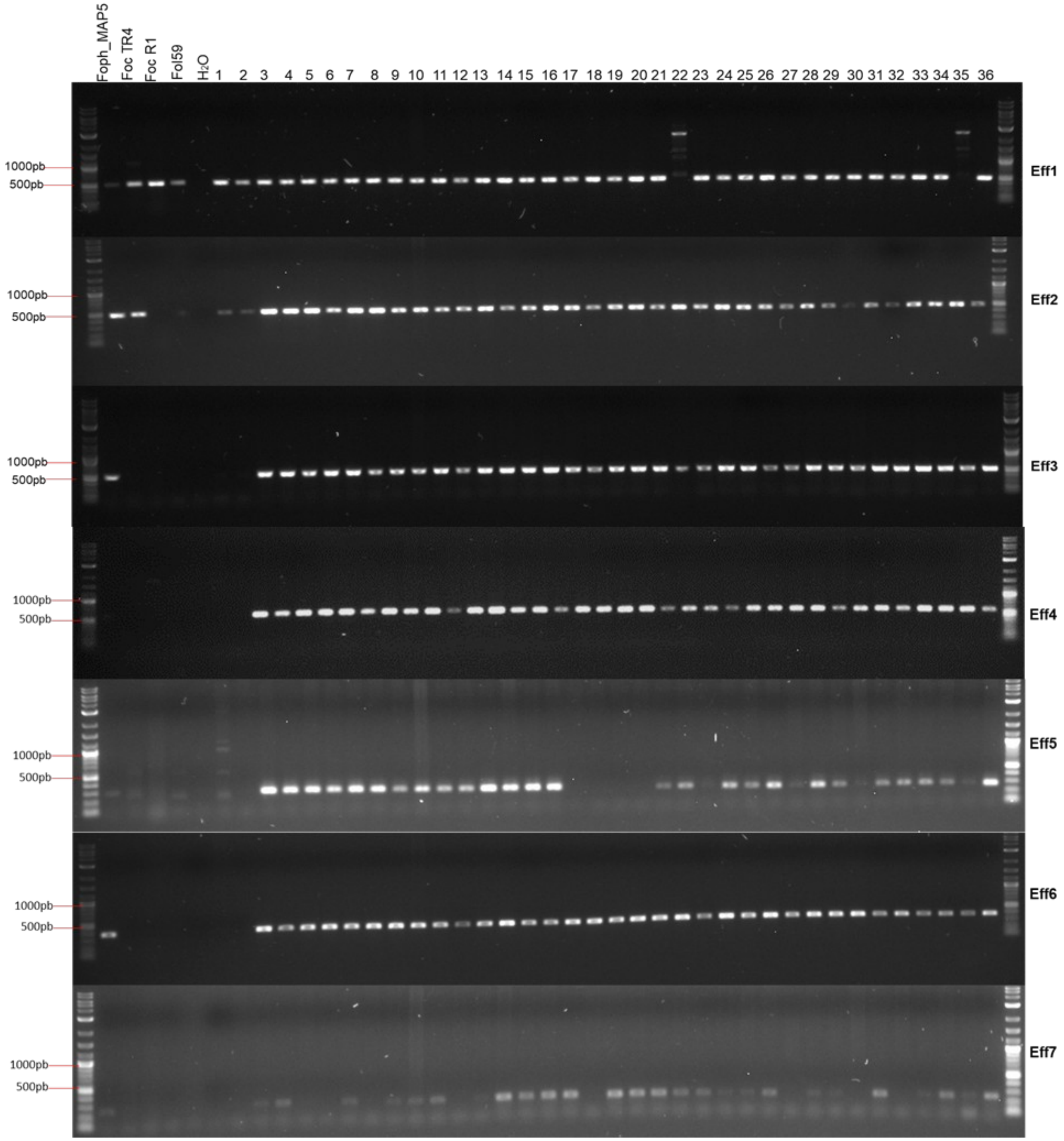
Foph_MAP5
Foc TR4
Foc R1
Fol59
H2O
1 2 3 4 5 6 7 8 9 10 11 12 13 14 15 16 17 18 19 20 21 22 23 24 25 26 27 28 29 30 31 32 33 34 35 36
1000pb
500pb
Eff1
Eff2
Eff3
Eff4
Eff5
Eff6
Eff7

C.

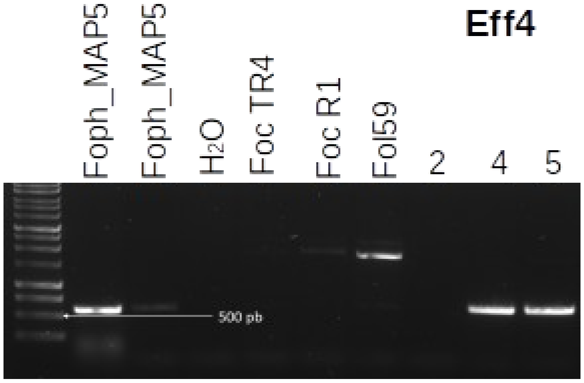

Figure S1. Confirmation of the novel *Foph* candidate effectors by PCR amplification **A.** Homologue of SIX13 **B.** Seven *Foph* novel candidate effectors (Eff1 to 7), showing their presence in a panel of 36 *F. oxysporum* isolates associated to the cape gooseberry crop (Table S2). **C.** Eff4 confirming its presence on *Foph*_MAP5. Control DNA: *Foph*_MAP5, Colombian isolates of Foc_TR4 (tropical race 4), Foc_R1 (race1) and Fol59. PCR product visualization was carried out following electrophoresis in a 1% agarose gel.
